# Introduction of a National Foundation Year 1 Mentoring Programme

**DOI:** 10.7759/cureus.93829

**Published:** 2025-10-04

**Authors:** Emma Whiting, Sagar Sanadi, Akash Doshi

**Affiliations:** 1 Medical Education, Queen Elizabeth Hospital Birmingham, Birmingham, GBR; 2 Emergency Medicine, Leeds Teaching Hospitals NHS Trust, Leeds, GBR; 3 Medicine, Barts Health NHS Trust, London, GBR

**Keywords:** foundation programme, medical education, mentee, mentor, mentoring, wellbeing

## Abstract

Background

The transition from medical student to Foundation Year 1 (FY1) doctor is marked by high stress and rapid professional adaptation, with many new graduates feeling underprepared. Mentoring has been shown to support resident doctors, but access to such programmes remains inconsistent across the United States.

Methods

In partnership with Mind the Bleep, a free medical education platform for resident doctors and healthcare professionals, a national, peer-led mentoring scheme was developed to support incoming FY1 doctors. The programme matched final-year medical students with current FY1 doctors across two cycles (August 2023 and August 2024 cohorts). Feedback was collected at the end of each cycle to assess engagement, perceived usefulness, and guide areas for improvement.

Results

The first cycle matched 420 mentees with 230 mentors (ratio 1.83), whilst the second cycle matched 861 mentees with 146 mentors (ratio 5.90). Although contact frequency varied, mentees reported benefits such as receiving advice and reducing anxiety, whilst mentors valued the opportunity to give back and reflect on their own experiences. Mean usefulness ratings ranged from 3.25 to 3.93 (out of 5). Participant feedback guided key areas for improvement including matching by geographical location and more structured guidance.

Conclusion

This national peer-led mentoring scheme offers accessible and valuable support to new FY1 doctors during a critical transition. The programme complements local efforts and demonstrates potential for sustainable, scalable impact across the NHS. According to the findings, it can be seen that the mentoring scheme demonstrated clear demand and perceived value amongst both mentors and mentees. The voluntary, peer-led model has proven feasible and sustainable with limited administrative input. Matching participants by location enhanced relevance but introduced logistical constraints. Feedback underscored a desire for more structured support and one-to-one mentoring, which are being addressed through enhanced mentor recruitment strategies and refined supporting materials.

## Introduction

The transition from medical student to Foundation Year 1 (FY1) doctor is widely recognised as a period of intense learning, psychological stress, and professional adjustment. Numerous studies have reported that many new graduates feel underprepared for clinical practice [1,2], with up to 25% of FY1 doctors experiencing burnout within their first year of work [3].

Mentorship has been shown to improve the wellbeing of resident doctors, enhance confidence, and contribute to professional development [4-6]. While several local mentoring programmes have been implemented across various hospital trusts [7,8], access to these schemes remains inconsistent and dependent on location or institutional resources. As such, many FY1 doctors are left without mentorship during this critical period.

In response to this gap, we designed and implemented a nationally accessible, peer-led mentoring programme for incoming FY1 doctors. This initiative was delivered in partnership with Mind the Bleep (MTB), a well-established medical education platform run by resident doctors [9]. By connecting final-year medical students (mentees) with current FY1 doctors transitioning to FY2 (mentors), the programme sought to provide guidance and pastoral support to new doctors as they enter clinical practice. 

Aims and objectives

The aim of this project was to establish and evaluate a sustainable peer-led mentoring programme to support the transition from medical student to FY1 doctors across the United Kingdom.

The objectives were to: (i) establish a scalable mentoring scheme, connecting final-year medical students with current FY1 doctors transitioning to FY2, (ii) evaluate the impact of the mentoring programme on the wellbeing of FY1 doctors and ease of transition to clinical practice, and (iii) identify potential areas of improvement through participant feedback, thus refining the design and delivery of the programme for future iterations.

## Materials and methods

The first cycle of the mentoring scheme targeted final year medical students about to become FY1 doctors in August 2023. Online sign-up forms were created using Google Forms (Google LLC, Mountain View, California, United States). The scheme was advertised, and sign-up forms were sent via established MTB Facebook and WhatsApp (Meta Platforms, Inc., Menlo Park, California, United States) groups, with the sign-up period running from June 8, 2023, to June 25, 2023.

Mentees were allocated to mentors based on geographical region, where possible, with the remaining participants allocated randomly. Contact details of the participants’ respective mentors/mentees were emailed on July 4, 2023, and all participants were encouraged to establish contact. When we were informed of participants dropping out or being unable to establish contact, participants were re-allocated. Feedback forms (see Appendices) were created using Google Forms and distributed to all participants in November 2023. Certificates of participation were provided following completion of the feedback form if an email address was provided; however, there was also an option to leave anonymous feedback. Feedback from the first cycle informed changes in the subsequent cycle.

The second cycle targeted the August 2024 cohort of new FY1 doctors and followed a similar design. The sign-up window ran from May 15, 2024, to May 26, 2024. Allocations were made primarily based on the location (deanery or hospital trust) of participants, with an option for participants to withdraw if a location match was not possible. Contact details of mentor/mentee allocations were emailed to participants on June 20, 2024, with a document containing expectations of the mentoring scheme, frequently asked questions, and helpful resources. An email was sent to all participants after two months, encouraging continued engagement between mentees and mentors and signposting to resources. Feedback forms were distributed to all participants in November 2024. Certificates of participation were provided following completion of the feedback form.

Statistical analysis

Data from feedback forms were exported from Google Forms into Microsoft Excel 2021 (Microsoft Corporation, Redmond, Washington, United States). Statistical analyses were performed using IBM SPSS Statistics for Windows, version 30 (IBM Corp., Armonk, New York, United States). Free-text responses were grouped thematically for analysis. Descriptive statistics were used to summarise demographic data and feedback responses. Categorical variables, such as frequency of contact, were presented as counts and percentages. Discrete variables, such as usefulness scores, were summarised using means and standard deviations.

Comparative analysis between the two cycles was conducted. Chi-square tests of independence were used to compare categorical variables between cycle 1 and 2, such as frequency of contact and reported benefits. When cell counts were less than 5, Fisher’s exact test was used. Mann-Whitney U tests were used to compare non-parametric discrete data between cycle 1 and 2, such as usefulness scores. Statistical significance was set at p <0.05 for all comparisons.

## Results

A total of 420 mentees and 230 mentors signed up to the first cycle of the scheme. We were informed of six mentors and two mentees who dropped out of the scheme or were not contactable, requiring reallocation. This resulted in 418 mentees paired with 224 mentors (ratio of mentees to mentors, 1.87). Each mentor was allocated between one and three mentees, with 190 out of 224 groups (84.8%) comprising one mentor and two mentees.

In the second cycle, 941 mentees and 146 mentors initially signed up. A total of 80 potential mentees withdrew from the scheme, for reasons such as pairing within their hospital trust not being possible. This resulted in 861 mentees participating (ratio of mentees to mentors, 5.90). Each mentor was allocated between two and 10 mentees, with the most common group size of five mentees (65 out of 146 groups (44.5%)).

A total of 47 mentors and 20 mentees completed feedback forms following the first cycle, whilst 29 mentors and 16 mentees completed feedback forms following the second cycle. Throughout both cycles, most participants contacted their mentee/mentor ‘less than monthly’ (33 out of 67 responses (49.3%) in cycle 1 and 19 out of 45 responses (42.2%) in cycle 2). Thirty responses (44.8%) and 19 responses (42.2%) in the first and second cycles, respectively, reported more frequent contact. Four responses (6.0%) and seven responses (15.6%) in the first and second cycles, respectively, did not establish contact (Table 1). A Chi-square test revealed no significant difference in the frequency of contact between cycles (χ^2^ (5) = 8.90, p = 0.113). A total of 14 participant responses (20.9%) in the first cycle and 15 participant responses (33.3%) in the second cycle reported difficulty contacting their mentor or mentees (Table 2). Fisher’s exact test indicated no statistically significant difference in difficulty establishing contact between cycle 1 and cycle 2.

**Table 1:** Frequency of reported contact between mentor and mentee in feedback form responses. Chi-square test of independence: χ^2^ (5) = 8.90, p = 0.113 (p > 0.05).

Frequency	Cycle 1 (n = 67), n (%)	Cycle 2 (n = 45), n (%)
Daily	0 (0.0)	1 (2.2)
Weekly	3 (4.5)	3 (6.7)
Fortnightly	6 (9.0)	8 (17.8)
Monthly	21 (31.3)	7 (15.6)
Less than monthly	33 (49.3)	19 (42.2)
Never	4 (6.0)	7 (15.6)
p-value	0.113	

**Table 2:** Frequency of reported difficulties contacting the mentor/mentee from feedback form responses. Fisher’s exact test: p = 0.22 (p > 0.05).

	Cycle 1, n (%)	Cycle 2, n (%)
Mentee Responses	2 (10.0)	6 (37.5)
Mentor Responses	12 (25.5)	9 (31.0)
Overall	14 (20.9)	15 (33.3)
p-value	0.22	

When asked to rate the usefulness of the mentoring scheme between 1 and 5 (where 1 is ‘not useful at all’ and 5 is ‘very useful’), the mean response for mentees was 3.75 (SD = 1.18) and 3.25 (SD = 1.56) in the first and second cycles, respectively. The mean response for mentors was 3.53 (SD = 1.20) and 3.93 (SD = 0.74) in the first and second cycles, respectively (Table 3). Mann-Whitney U tests did not reveal a statistically significant difference in usefulness scores between cycle 1 and cycle 2 for mentees (U = 185, p = 0.42) or mentors (U = 570, p = 0.22). Mentees most commonly reported that the scheme was beneficial for receiving general advice (12 responses (60.0%) in cycle 1; six responses (37.5%) in cycle 2) and easing anxiety (five responses (25.0%) in cycle 1; three responses (18.8%) in cycle 2). A Chi-square test of independence revealed no statistically significant difference between the reported benefits for mentees in cycle 1 and cycle 2 (χ^2^ (2) = 0.05, p = 0.98). Mentors most commonly reported that the scheme was beneficial for giving advice (20 responses (42.6%) in cycle 1; 10 responses (41.7%) in cycle 2), feeling they were helping others (11 responses (23.4%) in cycle 1), and reflecting on their own FY1 year (nine responses (19.1%) in cycle 1; two responses (8.3%) in cycle 2). A Chi-square test of independence revealed no statistically significant difference between the reported benefits for mentors in cycle 1 and cycle 2 (χ^2^ (4) = 8.69, p = 0.07) (Table 4).

**Table 3:** Reported usefulness of the mentoring scheme from feedback form responses, where 1 is ‘not useful at all’ and 5 is ‘very useful’. Data represented as n (%) and mean ± SD. Mann-Whitney U test to compare usefulness for mentees between cycle 1 and 2: U = 185, p = 0.42 (p > 0.05). Mann-Whitney U test to compare usefulness for mentors between cycle 1 and 2: U = 570, p = 0.22 (p > 0.05).

Usefulness	Cycle 1		Cycle 2	
	Mentees (n = 20)	Mentors (n = 47)	Mentees (n = 16)	Mentors (n = 29)
1	1 (5.0)	4 (8.5)	4 (25.0)	0 (0.0)
2	2 (10.0)	4 (8.5)	2 (12.5)	0 (0.0)
3	5 (25.0)	14 (29.8)	0 (0.0)	9 (31.0)
4	5 (25.0)	13 (27.7)	6 (37.5)	13 (44.8)
5	7 (35.0)	12 (25.5)	4 (25.0)	7 (24.1)
Mean	3.75 ± 1.18	3.53 ± 1.20	3.25 ± 1.56	3.93 ± 0.74
p-value			0.42	0.22

**Table 4:** Reported benefits of the mentoring scheme from feedback form responses. Chi-square test of independence for mentee responses across cycle 1 and cycle 2: χ^2^ (2) = 0.05, p = 0.98 (p > 0.05). Chi-square test of independence for mentor responses across cycle 1 and cycle 2: X2 (4) = 8.69, p = 0.07 (p > 0.05).

Benefit	Cycle 1		Cycle 2	
	Mentees, n (5)	Mentors, n (%)	Mentees, n (%)	Mentors, n (%)
Receiving general advice	12 (60.0)	0 (0.0)	6 (37.5)	0 (0.0)
Receiving specific job/hospital advice	2 (10.0)	0 (0.0)	1 (6.25)	0 (0.0)
Easing anxiety/having someone to talk to	5 (25.0)	0 (0.0)	3 (18.8)	0 (0.0)
Giving advice	0 (0.0)	20 (42.6)	0 (0.0)	10 (41.7)
Helping others	0 (0.0)	11 (23.4)	0 (0.0)	0 (0.0)
Reflection on FY1 year	0 (0.0)	9 (19.1)	0 (0.0)	2 (8.3)
Teaching and mentoring skills	0 (0.0)	3 (6.4)	0 (0.0)	4 (16.7)
Portfolio development	0 (0.0)	1 (2.1)	0 (0.0)	1 (4.2)
p-value			0.98	0.07

Following the first cycle, the most common suggestion for improvement was matching participants by location (21 responses (31.3%)), followed by increased guidance and prompts from the organisers during the scheme (12 responses (17.9%)), improving engagement between mentors and mentees (seven responses (10.4%)) and sending allocations earlier (seven responses (10.4%)). These suggestions were implemented in the second cycle. Following the second cycle, the most common suggestion for improvement was improving engagement between mentors and mentees (nine responses (20.0%)), followed by increased guidance and prompts from the organisers during the scheme (seven responses (15.6%)) and matching participants by location (four responses (8.9%)). A Chi-square test of independence suggested a statistically significant difference between the improvement suggestions in cycle 1 and cycle 2 (χ^2^ (6) = 14.46, p = 0.03) (Table 5).

**Table 5:** Suggestions for improvement to the mentoring scheme from feedback form responses. Chi-square test of independence: χ^2^ (6) = 14.46, p = 0.03 (p < 0.05).

Improvement Suggestion	Cycle 1, n (%)	Cycle 2, n (%)
Match by location / jobs	21 (31.3)	4 (8.9)
More guidance / prompts from organisers	12 (17.9)	7 (15.6)
Improved engagement of participants	7 (10.4)	9 (20.0)
Set up scheme / send allocations earlier	7 (10.4)	0 (0.0)
Ensure up-to-date contact details of participants	3 (4.5)	2 (4.4)
One-to-one mentoring, rather than groups	1 (1.5)	2 (4.4)
Provide more information about participants	0 (0.0)	1 (2.2)
p-value	0.03	

## Discussion

This national mentoring scheme has been positively received by both mentees and mentors, with feedback indicating a range of personal and professional benefits. Mentees reported that the programme provided valuable general advice and pastoral support, helping to alleviate anxiety during a stressful transition. Mentors, meanwhile, appreciated the opportunity to give back, reflect on their own FY1 experiences and develop teaching and mentoring skills, with additional benefits for portfolio development.

Although the number of feedback responses was limited, especially for mentees, we used them to guide improvements to the scheme. We attempted to encourage feedback responses by offering certificates of participation following completion. However, we recognise that mentors have more incentive than mentees to obtain a certificate to fulfil portfolio requirements. 

In response to feedback from the first cycle, we adapted the matching process to prioritise geographical location and hospital trust in an effort to facilitate more specific, context-relevant support, as geographical fluctuations are a known barrier to effective mentorship [10]. However, this change introduced logistical challenges: difficulty in matching based on location led to an imbalance in mentor and mentee allocations, with some mentors supporting up to 10 mentees. This stretched capacity may have limited the depth of interaction and reduced opportunities for individualised mentorship [11]. Looking ahead, we recognise the need to strike a balance between national accessibility and local relevance. While local mentoring schemes remain inconsistent across hospital trusts [7,8,12], this national programme fills an important gap. It offers fair access to mentorship regardless of location, while ideally complementing local initiatives where available. To strengthen its impact, future iterations will reinforce the scheme’s primary purpose: to offer general, non-location-dependent support during the early transition into clinical practice.

The voluntary, peer-led structure of the programme, run by a small team of resident doctors, has proven feasible and sustainable, with most administrative burden focused on participant allocation and periodic check-ins. The flexible design, lacking rigid timelines or requirements, allowed participants to engage on their own terms [10,11]. Whilst this approach supports autonomy, some participants indicated a need for clearer guidance on expectations and conversation structure. To address this, future cycles will include a more comprehensive handbook to enhance engagement and consistency.

A persistent challenge has been the imbalance between mentor and mentee sign-ups. Understandably, incoming FY1s may be more likely to seek support during this high-stress period, whilst current FY1s may feel less able to commit time as mentors. This has resulted in a group-based mentoring structure, which can dilute personal relationships and increase pressure on mentors [13]. Feedback from participants suggested a preference for one-to-one mentoring. To address this, we are considering strategies to increase mentor recruitment and retention, such as formal recognition of involvement, provision of mentorship training, and alignment with teaching and portfolio development goals, all of which are in line with effective mentorship [10,11,14].

Despite these limitations, the overall response to the programme has been encouraging. Participants consistently rated the scheme as useful, and qualitative feedback supports its continued value. The voluntary and informal nature of the initiative contributes to its accessibility and low-pressure environment: qualities that are particularly valuable during the stressful period surrounding the FY1 transition [15]. Supporting resident doctors is crucial to their overall wellbeing and, in turn, patient care [16]. Therefore, we will continue to run the mentoring programme in subsequent years.

## Conclusions

This national mentoring scheme has demonstrated feasibility and perceived value among both mentees and mentors. By offering accessible peer support across the United Kingdom, it fills an important gap in transitional support for new doctors. As we continue to refine the programme through participant feedback, we aim to enhance its structure, improve engagement, and promote sustainability. Ultimately, we envision this initiative to complement local wellbeing and mentorship efforts, supporting the professional and emotional wellbeing of FY1 doctors across the NHS.

## Appendices

Feedback form questions

1. What is your role in the mentoring scheme?

I am a mentee (current FY1)

I am a mentor (current FY2+)

2. On average, how often have you met/spoken to your mentor/mentee?

Daily 

Weekly

Fortnightly 

Monthly 

Less than monthly 

Never 

3. Did you experience any difficulties contacting your mentor/mentee? Please explain if so.

4. What have you found the mentoring scheme beneficial for?

5. How could the mentoring scheme be improved?

6. Overall, how useful did you find the mentoring scheme, on a scale of 1-5?

1 - not useful at all 

2 

3 

4 

5 - very useful 

7. Any other comments?

8. If you would like confirmation of participation, please enter your email address (optional)

9. If you would like confirmation of participation, please enter your name (optional)
